# Enhancing protein trapping efficiency of graphene oxide-polybutylene succinate nanofiber membrane via molecular imprinting

**DOI:** 10.1038/s41598-023-42646-5

**Published:** 2023-09-16

**Authors:** Nuankanya Sathirapongsasuti, Anuchan Panaksri, Benjabhorn Jusain, Sani Boonyagul, Suejit Pechprasarn, Kittisak Jantanasakulwong, Acharee Suksuwan, Somprasong Thongkham, Nuttapol Tanadchangsaeng

**Affiliations:** 1 Program in Translational Medicine, Faculty of Medicine Ramathibodi Hospital, Mahidol University, Ratchathewi, Bangkok, Thailand; 2 Chakri Naruebodindra Medical Institute, Faculty of Medicine Ramathibodi Hospital, Mahidol University, Bang Pli, Samut Prakan Thailand; 3 College of Biomedical Engineering, Rangsit University, Lak Hok, Pathumthani Thailand; 4 School of Agro-Industry, Faculty of Agro-Industry, Chiang Mai University, Mae Hia, Chiang Mai, Thailand; 5 National Nanotechnology Center (NANOTEC), National Science and Technology Development Agency (NSTDA), Klong Luang, Pathumthani Thailand; 6 The Halal Science Center, Chulalongkorn University, Pathum Wan, Bangkok, Thailand

**Keywords:** Biomaterials - proteins, Biopolymers

## Abstract

Filtration of biological liquids has been widely employed in biological, medical, and environmental investigations due to its convenience; many could be performed without energy and on-site, particularly protein separation. However, most available membranes are universal protein absorption or sub-fractionation due to molecule sizes or properties. SPMA, or syringe-push membrane absorption, is a quick and easy way to prepare biofluids for protein evaluation. The idea of initiating SPMA was to filter proteins from human urine for subsequent proteomic analysis. In our previous study, we developed nanofiber membranes made from polybutylene succinate (PBS) composed of graphene oxide (GO) for SPMA. In this study, we combined molecular imprinting with our developed PBS fiber membranes mixed with graphene oxide to improve protein capture selectivity in a lock-and-key fashion and thereby increase the efficacy of protein capture. As a model, we selected albumin from human serum (ABH), a clinically significant urine biomarker, for proteomic application. The nanofibrous membrane was generated utilizing the electrospinning technique with PBS/GO composite. The PBS/GO solution mixed with ABH was injected from a syringe and transformed into nanofibers by an electric voltage, which led the fibers to a rotating collector spinning for fiber collection. The imprinting process was carried out by removing the albumin protein template from the membrane through immersion of the membrane in a 60% acetonitrile solution for 4 h to generate a molecular imprint on the membrane. Protein trapping ability, high surface area, the potential for producing affinity with proteins, and molecular-level memory were all evaluated using the fabricated membrane morphology, protein binding capacity, and quantitative protein measurement. This study revealed that GO is a controlling factor, increasing electrical conductivity and reducing fiber sizes and membrane pore areas in PBS-GO-composites. On the other hand, the molecular imprinting did not influence membrane shape, nanofiber size, or density. Human albumin imprinted membrane could increase the PBS-GO membrane’s ABH binding capacity from 50 to 83%. It can be indicated that applying the imprinting technique in combination with the graphene oxide composite technique resulted in enhanced ABH binding capabilities than using either technique individually in membrane fabrication. The suitable protein elution solution is at 60**%** acetonitrile with an immersion time of 4 h. Our approach has resulted in the possibility of improving filter membranes for protein enrichment and storage in a variety of biological fluids.

## Introduction

Protein analysis is an important technique in various fields, such as biochemistry, medicine, biotechnology, and environmental science^1,2^. Accurate identification of protein types and precise measurement of protein quantities are critical for understanding biological processes, diagnosing diseases, and assessing the quality of food and drugs^3–7^. However, analyzing proteins in biological fluids is challenging due to the low abundance, high molecular weight, and susceptibility to degradation of target proteins^8,9^. Moreover, it is necessary to separate target proteins from the fluid for specific protein analysis. Chromatography and electrophoresis are commonly used techniques for protein separation based on the behavior of proteins during fluid flow and electric migration, respectively^10,11^. However, these techniques are complex, time-consuming, and expensive, which is unsuitable for analyzing many protein samples or randomly collected protein samples^12,13^. Therefore, there is a need for convenient and straightforward methods for protein or biomolecule separation and analysis.

Membrane filtration is a technique that has gained attention for protein separation due to its ease of use, lack of energy requirements, and absence of additives used to trap proteins^14,15^. Membrane filters trap molecules based on size, with molecules larger than the membrane’s pore size being blocked^16,17^. The use of membrane filters is applied in combination with the syringe-push membrane absorption (SPMA) technique to study the method of reducing the steps of protein storage in liquid samples^18^. SPMA is a filtration method for liquid samples through a membrane filter under pressure from a syringe. This method accelerates the suspended particles to attach to the membrane filter faster. Currently, improvements in membrane filter technology aim to match the pore size to the target molecule, with nanofiber membranes created using electrospinning being widely studied^19^. These membranes have a high surface area, tunable pore sizes, and high strength, making them suitable for capturing small biomolecules^20,21^. However, protein capture using membrane filters still has a significant limitation regarding specificity for the target molecule. As membrane filters capture molecules based on their molecular size, high concentrations of non-target molecules can interfere with the capture efficiency of the target molecule^22,23^. Previous studies have used polybutylene succinate nanofiber membranes mixed with graphene oxide to increase the efficiency of protein capture. However, the protein capture efficiency was only 40%, even with the addition of graphene oxide. Although the performance obtained from the nanographene oxide membrane is higher than conventional membrane filtration by up to 2 times, this performance figure indicates that the previous study’s graphene oxide membrane could not filter out all proteins^24^. The study suggests a reduction in the specificity of protein binding to the membrane, resulting in decreased filtration efficiency.

Currently, an interesting technique can be used to enhance the efficiency of protein capture called “Molecular Imprinting”. This technique involves creating a biological recognition element at the molecular level on a material, predominantly a polymer, to capture a specific molecular target^25,26^. Essentially, molecular imprinting creates a lock-and-key mechanism for the target molecule. This technique can increase the specificity of protein capture and has been applied in various fields, such as creating biosensors and measuring biological substances^27,28^. Using the imprinting technique to improve protein capture specificity in memory-based assays shows promising potential for enhancing the efficiency of protein capture.

**Figure 1:**
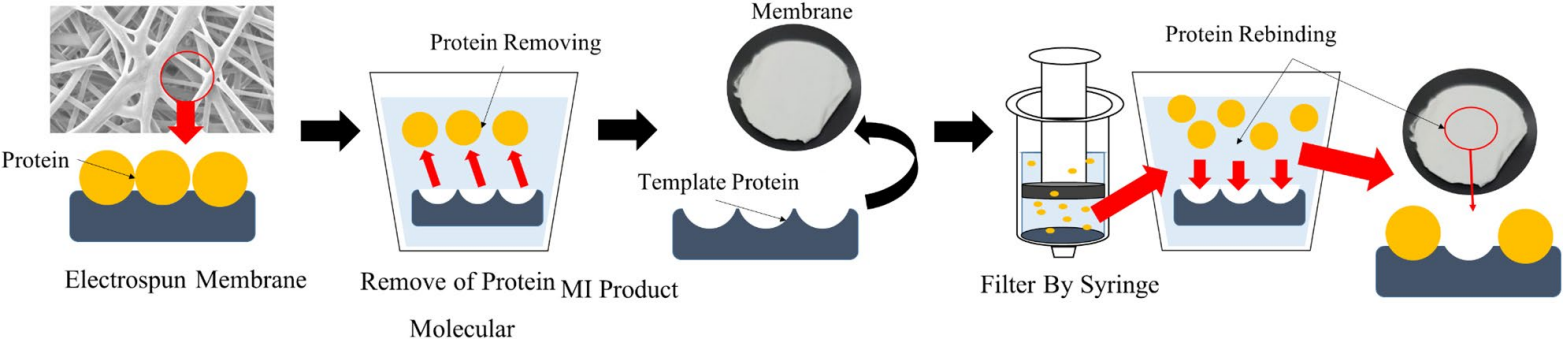
Molecular imprinting process performed on the nanofibrous membrane using albumin protein as a template molecule as well as the removal of the template protein, resulted in molecular-level memory.

This study utilized the imprinting technique to improve the performance of a protein absorption filter membrane. The filter membrane was created using the electrospinning technique with bioplastics polybutylene succinate and composite with graphene oxide to form the nanofibrous membrane. The imprinting process was performed on the nanofibrous membrane using albumin protein as a template molecule, and the removal of the template protein resulted in molecular-level memory, as depicted in the schematic in Fig. 1. The imprinted nanofibrous membrane was characterized and evaluated for its protein trapping ability, high surface area, potential for generating affinity with proteins, and molecular-level memory. These features may enhance the protein trapping performance of the membrane filter, which can be beneficial for storing and reducing protein losses in liquid samples and for analyzing various biological processes in the future.

## Materials and methods

### Materials

Polybutylene succinate (PBS) (FD92) was supplied by PTT MCC BIOCHEM CO., LTD. (Bangkok, Thailand). Graphene oxide powder, 15–20 sheets, 4–10% edge-oxidized, was purchased from Sigma-Aldrich (St. Louis, MO, USA). Albumin from human serum (ABH) and fetal bovine serum albumin (FBS) were purchased from Sigma-Aldrich, St. Louis, MO, USA. Hexafluoro-2-propanol (HFIP) was purchased from Sigma-Aldrich (St. Louis, MO, USA). Brilliant Blue Coomassie G-250 was purchased from ACROS Organics™. Acetonitrile was purchased from RCI Labscan (Bangkok, Thailand).

### Solution preparation for electrospun fiber molecular imprinting membrane

The solvent preparation was divided into a solvent group for non-composite graphene oxide imprinting membrane and a solvent group for composite graphene oxide imprinting membrane. Both solutions were prepared by dissolving PBS (8 wt%) in hexafluoro-2-propanol solution, which was heated at 60 °C for 24 h. Once the PBS solution was obtained, the group of solutions without composites would be prepared for the next step. Meanwhile, the group of composite solutions was supplemented with 0.1% w/w of graphene oxide in PBS and mixed at 60 °C. Then, the solution containing the graphene oxide was undergo sonication for 15 min to obtain the solution of graphene oxide composites. The two groups of solutions were mixed with albumin, divided into two conditions: a condition mixed with human albumin (ABH) and a condition mixed with fetal bovine serum (FBS). Both solutions were cooled to room temperature and supplemented with 2 wt% albumin. After the solutions were homogenous, they were loaded into syringes for further processing.

### Fabrication of electrospun fiber molecular imprinting by electrospinning

A filter membrane was prepared using a single-nozzle electrospinning setup with a rotating drum collector (rolling electrospinning) at a speed of 500 rpm. A high-voltage power supply (ES60P-10 W, Gamma High Voltage, Ormond Beach, FL, USA) was connected to a metal nozzle to create a voltage difference of 25 kV between the metal nozzle and a collector. The charged metal nozzle used in the experiment had an outer diameter of 0.57 mm and was positioned 15 cm from the collector covered with aluminum foil. The solution was delivered from a plastic syringe to the metal nozzle at a rate of 3 mL/h using a mechanical pump (NE-300 Just Infusion™ Syringe Pump New Era Pump Systems, Inc., Farmingdale, NY, USA), and the feeding process took 4 h. The membrane obtained from the fabrication process was peeled off the aluminum foil to create molecular imprinting. Molecular imprinting on the membrane was performed by immersing the membrane in DI water for 4 h. The immersed membrane would have marks from the leaked-out albumin molecules on the nanofiber. The membranes soaked in DI water for 4 h were oven-dried at 40 °C for 24 h before use.

### Examination of morphology by scanning electron microscopy

The scanning electron microscope (SEM) (Quanta 250, FEI, Hillsboro, OR, USA) was used to analyze the morphology and size of the electrospun fiber samples, which were prepared in 2 × 2 cm^2^ dimensions. The electrospun membrane specimens were observed at a magnification of 10,000 times and an accelerating voltage of 12.5 kV. For the measurement of fiber diameter, as well as the determination of average fiber diameter and average pore area along with standard deviation (SD), ten readings were taken using Image J software (www.imagej.nih.gov, accessed on 1st May 2021). The pore size is determined using theoretical calculations presented in the form of a pore radius^29^. These calculations rely on equations as described in Eqs. (1) and (2).

1 $$Porosity (\varepsilon )=\frac{\mathrm{W}swollen-\mathrm{Wdry}}{\mathrm{d}\times \mathrm{A}\times\uprho }$$

where d is the membrane thickness (m), A is the area of the membrane (m^2^), and ρ is the density of the water (998 kg m^−2^). We then used the Guerout-Elford-Ferry equation to calculate the mean pore radius (rm)^30,31^.

2 $$Pore \,radius=\sqrt{\frac{(2.9-1.75\varepsilon )\times 8\eta dQ}{\mathrm{A}\times \Delta \mathrm{P}\times\upvarepsilon }}$$

where η is the viscosity of the water (8.9 × 10 − 4 Pa s), Q refers to the volumetric flux of the water (m^3^ s^−1^), and ΔP is the operating pressure (Pa).

### Mechanical properties pest of membranes

A stress–strain test of membranes (20 × 5 × 0.15 mm^3^) was performed at room temperature using an Instron bench-type tensile test machine (Instron 5943 universal testing system) with load cell at 50 N at a strain rate of 5 mm/min.

### FTIR measurement of electrospun fiber molecular imprinting membranes

The membrane samples were subjected to examination using the ATR-FTIR technique. Scanning was performed at a resolution of 4 cm^−1^ with 64 scans across a wavenumber range spanning from 400 to 4000 cm^−1^. This analysis was carried out using the FT-IR spectrometer (model: Nicolet 6700, equipped with a DLaTGS detector) from Thermo-Scientific, USA. The recorded IR spectra captured the characteristic peaks. The measurements were divided into three groups: Non-imprinting Membrane (PBS + GO), Imprinting Membrane, and Imprinting Membrane without elution proteins.

### Comparison of membrane protein binding capacity

Circular membranes with a diameter of 13 mm each (n = 3) were prepared for every individual condition. Subsequently, a solution containing human albumin at a concentration of 1 mg/mL was created using deionized (DI) water, and then 5 mL of this solution was filtered through each membrane using the SPMA technique. The membrane's ability to adsorb was determined by comparing the protein content traversed through it. To quantify the protein content, 10 μL of the effluent from each membrane was collected and subjected to analysis with a Bradford reagent kit (Bio-Rad), adhering to the guidelines provided by the manufacturer. The percentage of albumin proteins bound to the electrospun filter (% protein adsorption) was calculated using Eq. (1).

1 $$\% \;{\text{Protein adsorption }} = \, ((1 \, - x)/1) \times 100$$

where 1 is 100% albumin in the testing solution, and x is the albumin passed through the filter.

### Staining of protein on the filtered specimens

To perform protein staining, a Neuhoff dye solution comprising 2% H_3_PO_4_, 10% (NH_4_)_2_SO_4_, 20% methanol, and 0.1% Coomassie G-250 was utilized. Adding to the membrane already undergoing the SPMA technique, 200 μL of the dye solution was carefully pipetted. Following a 30-min incubation, the dye reagent was thoroughly washed away using deionized (DI) water. The filter samples were subsequently air-dried, and photographs were captured to assess the protein distribution on each filter membrane qualitatively. ImageJ software employed images of the stained membranes to gauge the intensity of the blue color present. The obtained intensity readings were then standardized by referencing the intensity value of the unstained membrane.

### Examination of the appropriate concentration for protein elution from the membrane

The acetonitrile concentration was determined for finding the appropriate concentration using 55, 60, 65, and 70% concentrations in the experiment. The selected membrane filters that passed through the protein solution filtration process were immersed in acetonitrile at the specified concentration for 4 h. After the 4-h immersion of the membrane in acetonitrile, the protein content was measured using the Bradford reagent kit (Bio-Rad) according to the manufacturer’s protocol.

### Examination of the appropriate time for protein elution from the membrane

The selected membrane was immersed in acetonitrile with 60% and 70% concentrations. Each membrane will be immersed for a different duration, ranging from 30 min to 8 h. After immersing the membrane, the acetonitrile solution was measured for protein quantification using the Bradford reagent kit (Bio-Rad) according to the manufacturer’s protocol.

### Statistical analysis

The analysis of the data was conducted utilizing SPSS Statistics software version 25.0 (IBM, USA) and Microsoft Excel 2019 (Microsoft, USA). To compare two sets of data, a paired t-test was employed. Statistically significant distinctions between means were acknowledged at the 95% confidence level (*p* < 0.05).

## Results and discussions

### Morphology of the fabricated membranes

**Figure 2:**
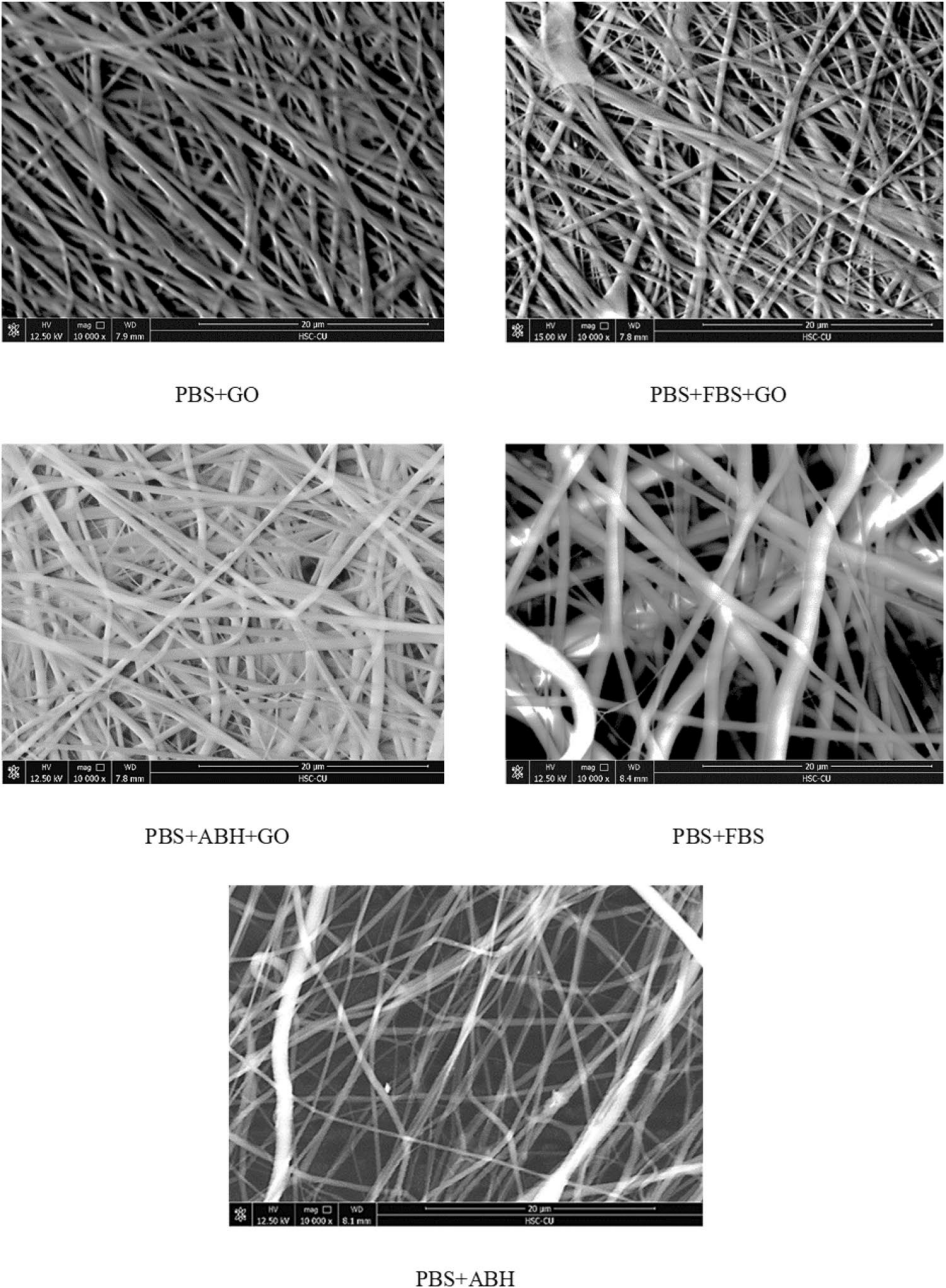
Morphology of electrospun fibers at 10,000 × magnification.

The morphological features of protein filter membranes were examined through an SEM, which revealed the characteristics of the membrane, as shown in Fig. 2. The observation at a magnification of 10,000 × showed the arrangement of nanofibers formed using electrospinning techniques at different conditions. PBS membrane of the imprinting group using albumin (human albumin (ABH) and fetal bovine serum (FBS)) without composite with graphene oxide (GO) shows a lower nanofiber density compared to the membrane PBS group that was composite with graphene oxide using both imprinting and non-imprinting techniques.

The apparent difference observed in Fig. 2 is the density of the nanofibers. The characteristic of the group of the membrane with low nanofiber density is the group that does not have any composites with graphene oxide, indicating that GO is a determinant for the characteristic of nanofibers that are sprayed out. The imprinted membranes and non-imprinted exhibit the same fiber density when composite with GO.

The diameter and pore size of the nanofibers were analyzed from SEM images taken at a magnification of 10,000×. The results of the Image J software analysis are shown in Table 1. The average centerline diameter of PBS + GO (non-imprinting), PBS + FBS, PBS + ABH, PBS + FBS + GO, and PBS + ABH + GO nanofibers were 0.87, 1.28, 1.10, 0.87, and 0.92 μm, respectively. The largest fibers are from the PBS + FBS group, while the PBS + ABH group has the next largest fibers, followed by the remaining groups with similar fiber sizes. The result of the examination of the pore radius of the membrane correlates with Fig. 2. The imprinted membranes mixed with graphene oxide (PBS + GO, PBS + FBS + GO, and PBS + ABH + GO) have pore radius in the range of 22 to 24 μm^2^, while the group of membranes that were not composite with graphene oxide have pore areas in the range of 30 to 36 μm^2^.

**Table 1:** Fiber diameter and membrane area from SEM images.

Sample	Average diameter of fibers (µm)	Average pore radius (µm)
PBS + GO	0.87 ± 0.17	5.34 ± 0.71
PBS + FBS	1.28 ± 0.32	8.47 ± 2.42
PBS + ABH	1.10 ± 0.28	7.12 ± 1.61
PBS + FBS + GO	0.87 ± 0.30	4.85 ± 0.96
PBS + ABH + GO	0.92 ± 0.78	5.14 ± 0.67

The consideration of the diameter of the nanofiber and the pore size shows differences that correspond to the SEM results. The group of the membrane that did not undergo graphene oxide treatment had a larger average diameter and pore area than those treated with graphene oxide, both in the imprinting and non-imprinting groups, as determined by the results of the previous study (PBS + GO)^24^. Graphene oxide is a substance that significantly affects the membrane’s morphology when considering the results from SEM and the size of nanofibers. The property of graphene oxide is its ability to enhance electrical conductivity, resulting in a more efficient transfer of nanofibers to the fiber collector^24,32^. The fibers obtained from the graphene oxide-composited will have smaller sizes and can be more efficiently deposited onto the nanofiber collector compared to the membranes without graphene oxide, acting as a better nanofiber conductor. Injecting higher-weight fibers without a suitable fiber carrier leads to larger fiber size and lower fiber density. When considering the imprinting technique of human blood group proteins (ABH) and animal serum albumin (FBS) compared to non-imprinting techniques that only use graphene oxide, it was found that both imprinting and non-imprinting techniques did not affect the size and density of nanofibers, because graphene oxide serves as a controlling factor in the morphology of the resulting membrane. Upon evaluating all the membranes, there are three conditions that yield similar results. These conditions include those who are not using the imprinting technique. However, the sample with differing fiber size is the one that involves membranes without GO, specifically membranes that were not mixed with GO. Therefore, the assumption is made that the variation in fiber size in comparison with other conditions is related to the presence of GO. This is because the MIPs produced using the imprinting technique with GO as a component do not exhibit a fiber size distinct from the original technique that excludes GO as a component. The higher fiber size observed in conditions PBS + ABH and PBS + FBS may be due to their lower capability for fiber alignment compared to MIPs produced from a polymer solution containing GO. GO is a conductive material, which may facilitate faster movement of electrical charges along the fiber when injected into the receptor base, potentially enhancing the alignment process.

### Tensile test of fabricated membranes

**Figure 3:**
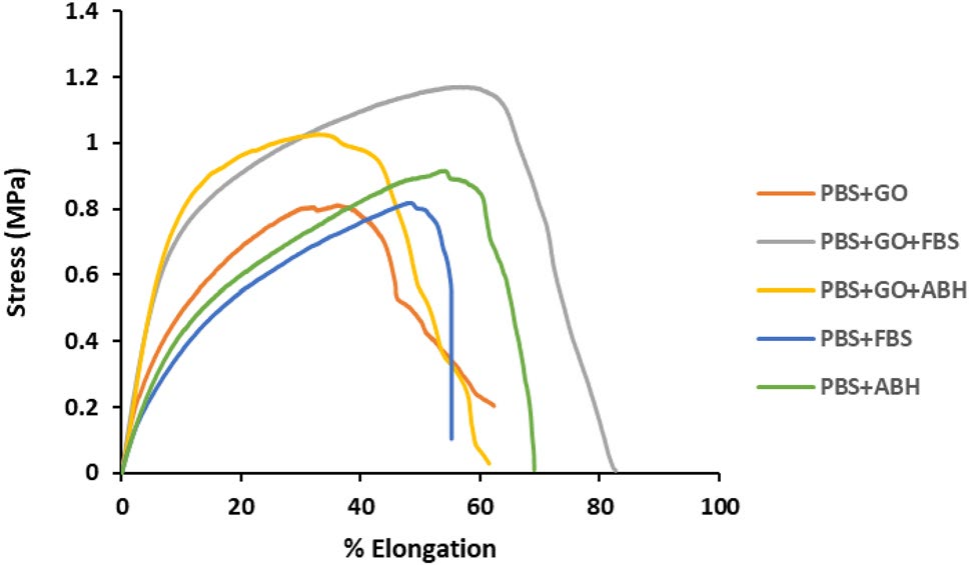
The stress–strain curves of the membrane samples for each condition.

The mechanical property testing of the membrane required the use of a low-load tensile testing machine due to the special thin nature of the membrane^33^. The load cell used for this testing was 10 N. The results of the mechanical properties examination of the membrane under different conditions are presented in Fig. 3, showing the stress–strain curve. Mechanical properties are summarized in Table 2. The results demonstrate differences in membrane tensile strength between groups with and without graphene oxide. Membranes PBS + FBS and Membrane PBS + ABH exhibit lower tensile stress compared to PBS + GO, PBS + GO + FBS, and PBS + GO + ABH. Hence, it can be inferred that graphene oxide enhances the membrane's strength. However, this study does not provide insight into the distribution of graphene oxide within the material. Considering the material's mechanical properties, it can be observed that graphene oxide significantly impacts the membrane's formation. Even though membranes that are composited with graphene oxide, both with and without imprinted patterns, show similar tensile strength values, these Membranes exhibit differences in % elongation. Membrane PBS + GO, which has lower percent of elongation than membranes in all molecular imprinting pattern groups, may be attributed to the fabrication technique that results in voids at the molecular level within the fiber, potentially affecting its stretching ability. Nevertheless, voids at the molecular level do not reduce the material's tensile strength and structural stability, possibly due to the dispersion of graphene within the fiber's components.

**Table 2:** Mechanical properties evaluation of membrane samples under each condition (n = 3).

Sample	Ultimate strength (MPa)	Elongation (%)	Young’s modulus (MPa)
PBS + GO	0.81 ± 0.27	36.07 ± 16.77	9.40 ± 3.54
PBS + FBS	0.50 ± 0.41	48.34 ± 7.30	5.54 ± 2.48
PBS + ABH	0.47 ± 0.35	55.41 ± 8.54	5.92 ± 3.15
PBS + FBS + GO	0.76 ± 0.22	63.77 ± 14.58	11.25 ± 2.14
PBS + ABH + GO	0.93 ± 0.13	40.20 ± 15.67	10.91 ± 3.25

### FTIR measurement of fabricated filter membranes

**Figure 4:**
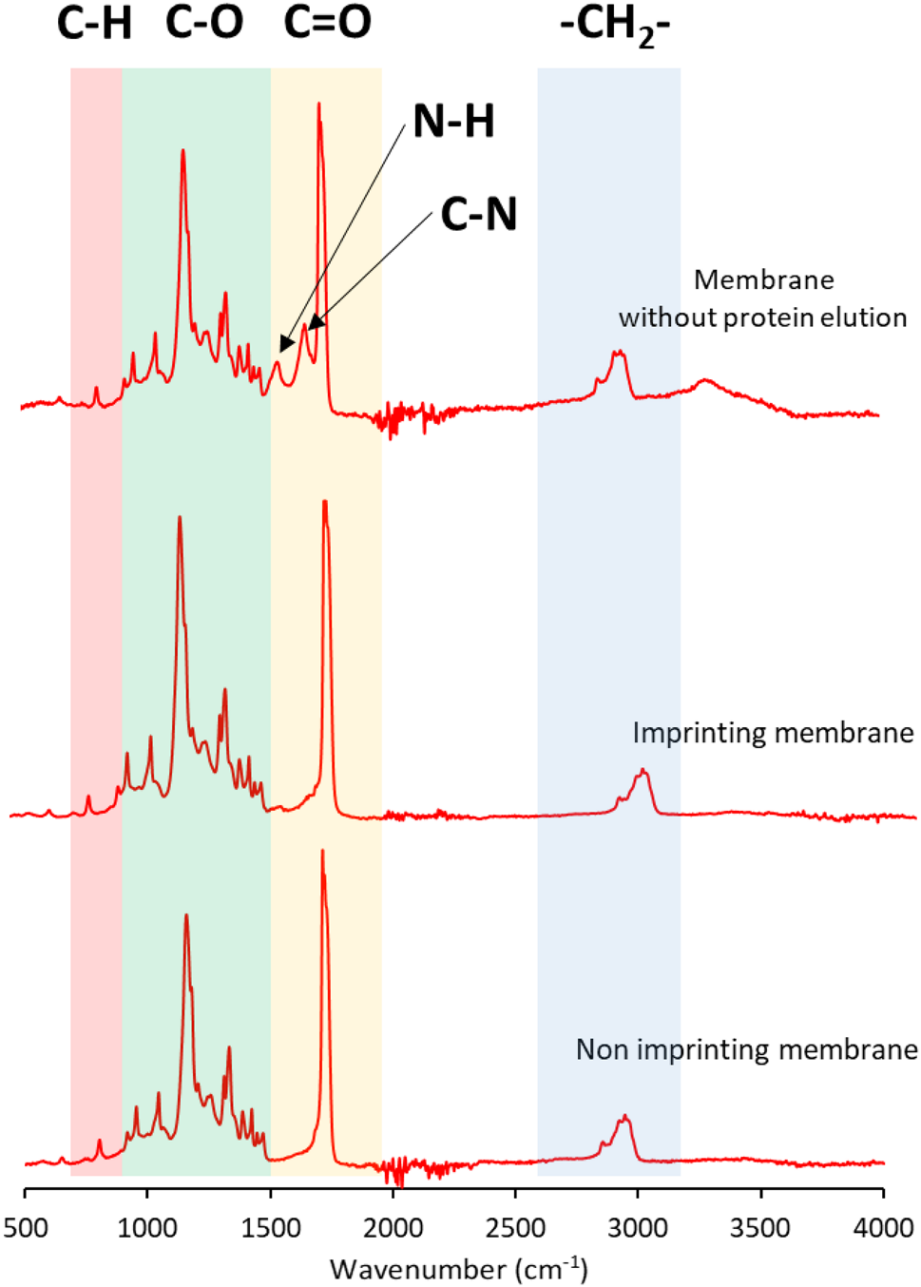
FTIR spectra of fabricated membrane samples.

The chemical functional groups of the membranes were examined and classified into three groups: Non-Imprinting Membrane (PBS + GO), Imprinting Membrane, and Imprinting Membrane without proteins, as shown in Fig. 4. The FTIR measurements revealed the general chemical functional groups present in PBS, including methylene groups (–CH_2_–) at 2945–2922 cm^−1^, carbonyl moiety (C=O) at 1712–1724 cm^−1^, ester moiety (C–O) at 1100–1200 cm^−1^, and alkyl moiety (C-H) at 600–700 cm^−1^. In addition to these chemical functional groups, the group of non-imprinted membranes showed the presence of amide groups, which consist of amide bands in two regions: N–H bending and C-N stretching at 1650–1660 cm^−1^ and 1550–1560 cm^−1^, respectively. However, no significant peaks corresponding to the aforementioned functional groups, such as amide bands, were observed in the imprinted membranes. This observation was consistent with the group of imprinted membranes without proteins. Consequently, it can be concluded that the imprinted membranes effectively removed proteins from the membranes, as evidenced by the absence of characteristic protein-related functional groups.

### The protein binding capacity of the fabricated filter membranes

**Figure 5:**
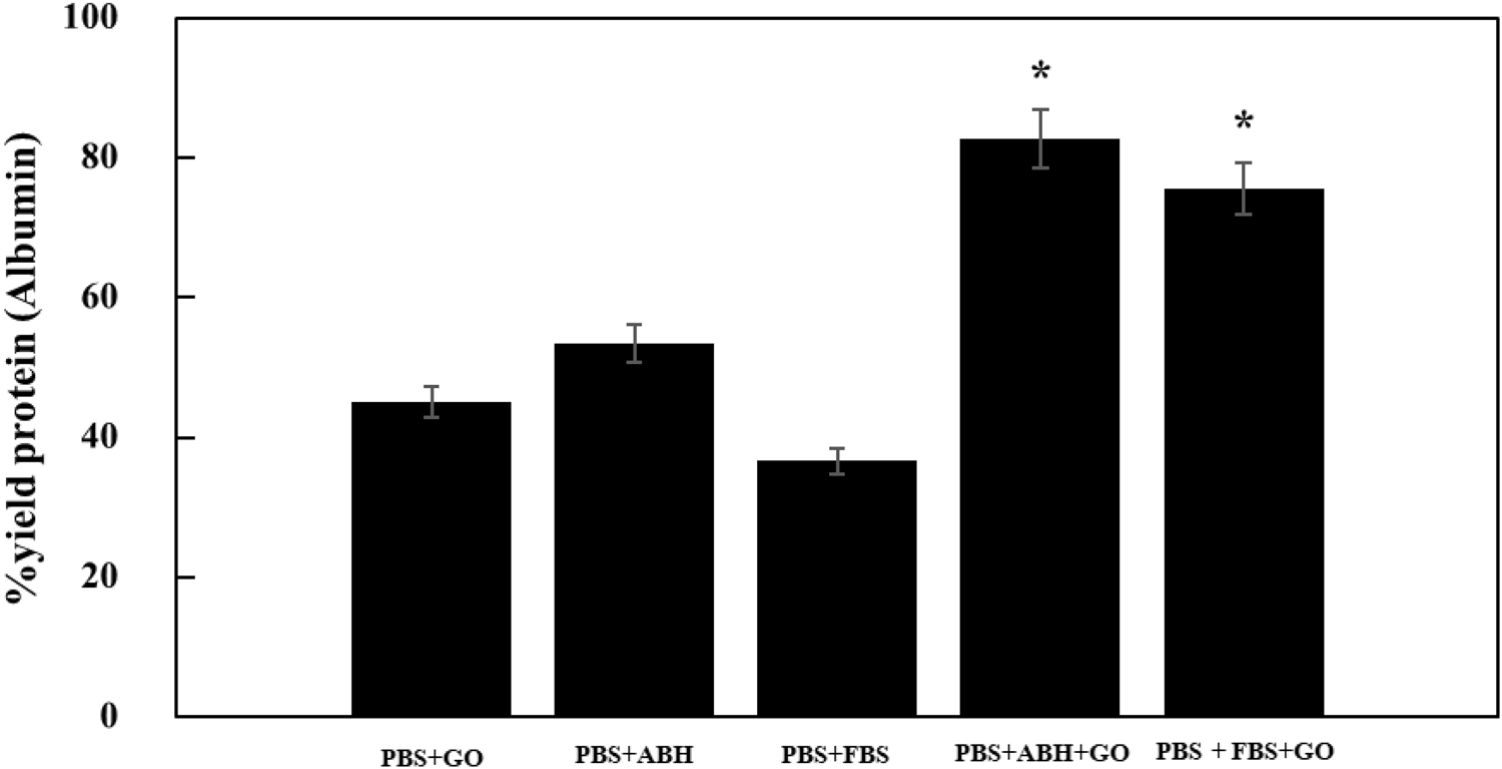
Yield protein of fabricated membranes (n = 3, *significant differences of the two samples compared to the others (p < 0.05)).

The ability to capture proteins was evaluated by measuring and calculating the amount of protein recovered from the liquid filtered through each membrane condition. Figure 5 shows the amount of protein that the membrane can capture. The membrane that can capture the most protein is PBS + ABH + GO, with a protein yield of approximately 83%. In contrast, PBS + FBS + GO has a slightly lower protein yield at approximately 76%. The two conditions of the highest protein capture efficiency on the membrane belong to the imprinted membranes with graphene oxide composites group. The evaluation of non-imprinting membrane groups (PBS + GO) and membrane groups without graphene oxide (PBS + FBS, PBS + ABH) found that the captured protein amount was within 40 to 50%. The results above demonstrate the difference in protein capture ability between the membrane with improved properties using the imprinting technique and the group without using the imprinting technique, which is also related to the factor of using graphene oxide.

The study examines the differences in protein capture ability depending on the conditions and techniques used to modify the membrane. The membrane group with the highest ability to capture the amount of albumin protein is the membrane group imprinted with albumin protein and GO. Human albumin (ABH) without adding graphene oxide showed slightly higher results than the previous study (PBS + GO)^24^. On the other hand, animal albumin (FBS) yielded results similar to the previous study. However, there was no statistically significant difference between these two conditions compared to the protein content obtained from the conventional membrane. Although graphene oxide imprinting dramatically enhances the ability of the membrane to capture proteins, when considering only membranes that undergo a specific technique, some of them exhibit similar protein-capturing ability regardless of whether or not they are imprinted with graphene oxide, with only half of the membranes imprinted and graphene oxide composited showing increased protein capturing ability. The protein amount of the reported membrane proteins indicates that the increased protein-capturing ability is due to the synergistic effect of two membrane protein modification techniques: surface imprinting and composites with graphene oxide. The surface imprinted membranes were also based on both albumin proteins' shape memory. During the surface imprinting process, the high surface/volume ratio graphene oxide acts as functional monomers to self-rearragement to interact with albumin proteins via hydrogen bonds, electrostatic forces, hydrophobic interactions, and π-π stacking effects^34,35^. The structural similarity of ABH (66.4 kDa, pI 4.7) and FBS (66.5 kDa, pI 4.5–5.0) leads to close protein absorption on surface imprinted membranes^36,37^. When considering membranes that utilize imprinting-only, it can be observed that the density of fibers in the membrane is lower than in membranes that utilize graphene oxide composite via the effect of using fewer fibers. The imprinting process, designed to serve as a template for capturing protein molecules, is created on nanofibers. Therefore, the low amount of nanofibers leads to less surface area for the template to interact with protein molecules, resulting in decreased efficiency of protein capture.

When considering the membranes in our previous study (PBS + GO electrospun fiber membrane without imprinting)^24^, it can be observed that the density of nanofibers is not different from those created with imprinting and graphene oxide composites. However, it has a significant effect on protein capture efficiency due to imprinting. Using graphene oxide as a biological recognition may not be sufficient for creating specific protein interactions. Studying the composite of graphene oxide could be significant in promoting the alignment of nanofibers to increase their density more than using them as protein capture agents. However, when considering both membrane techniques, it can be explained that using imprinting together with graphene oxide creates a membrane with efficiency in protein capture. Thus, it is necessary to use both techniques together to achieve the desired results. This study demonstrates that imprinting protein molecules can capture specific molecules with sufficient surface area with the imprinted pattern.

### Qualitative investigation of absorbed protein on the membrane by protein staining

**Figure 6:**
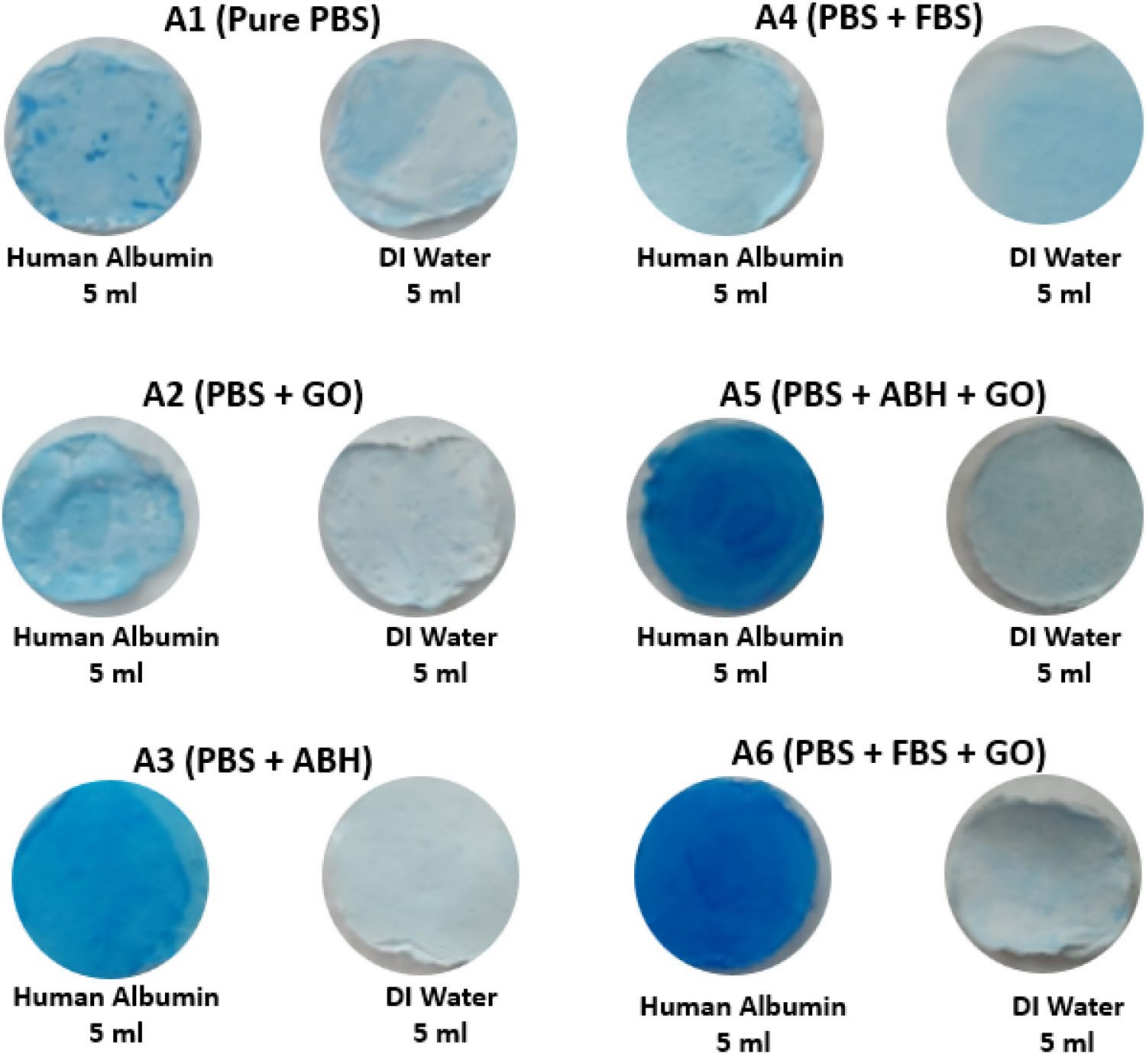
Brilliant blue Coomassie G-250 staining of absorbed proteins on membranes.

The ability to capture proteins was assessed qualitatively by demonstrating protein staining on the membrane using the Neuhoff dye staining protocol^38^. Figure 6 shows the membrane filtered with protein solutions and stained with blue dye, demonstrating different intensities compared to the membrane filtered with DI water, which is used to compare dye intensity levels. The staining results indicate that the highest level of staining intensity was observed in the group of membrane filters that underwent imprinting and graphene oxide treatment, particularly in the case of imprinting with human protein (ABH) and animal protein (FBS).

**Figure 7:**
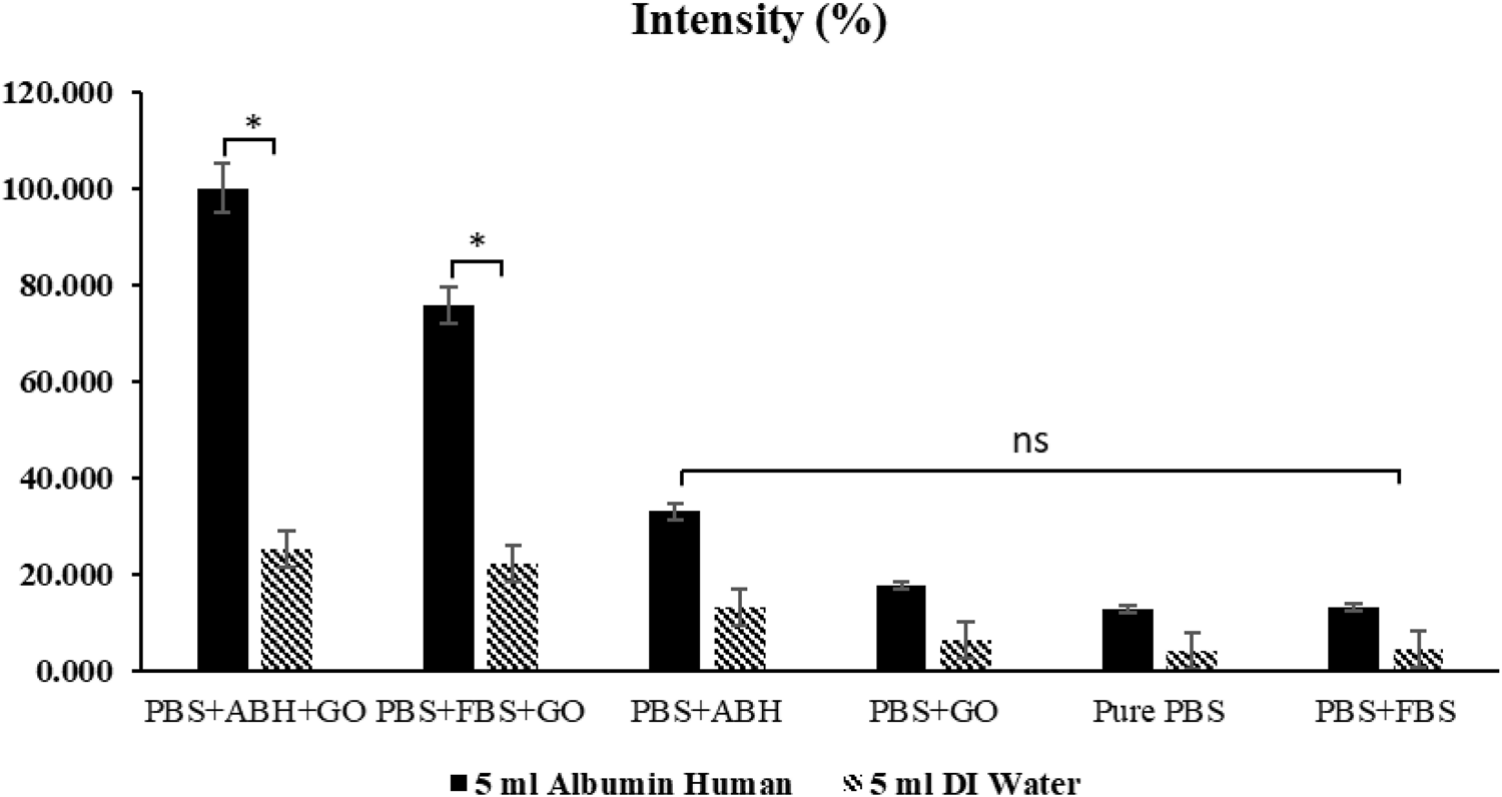
Intensity of staining membranes from ImageJ software (n = 3, ns = not significant, * significant differences of the two samples compared to the others. (p < 0.05)).

The membrane stained with dye indicates that proteins have been captured on the membrane. However, it should be noted that the quality of the staining on the membrane may have some variability in differentiating the intensity levels with the naked eye. Therefore, the staining data was confirmed by measuring the blue color intensity on the membrane using ImageJ software, which calculates the intensity of the stained image. The results of measuring the intensity using the image of the membrane filtered with DI water, which did not have any dye, served as a reference value to show the data related to the image. The intensity level of the imprinted and composited graphene oxide membranes had the highest value (A5 and A6), as shown in Fig. 7.

The brilliant blue staining on the membrane supports the protein binding results on the imprinted membrane and the graphene oxide composited, measured by back-calculation from the fluid filtered through the membrane. The dark blue color on the membrane indicates that the developed membrane has captured proteins^39^. The measurement of the intensity of the dye on the membrane corresponds to the quality data and the ability to capture proteins that were measured, which confirms the hypothesis that imprinting and composite graphene oxide are techniques that enhance the ability to capture proteins. Using only one technique may not be sufficient to capture proteins efficiently, but combining these techniques can improve protein capture ability.

### Influence of solution and immersion time on protein elution from membrane

**Figure 8:**
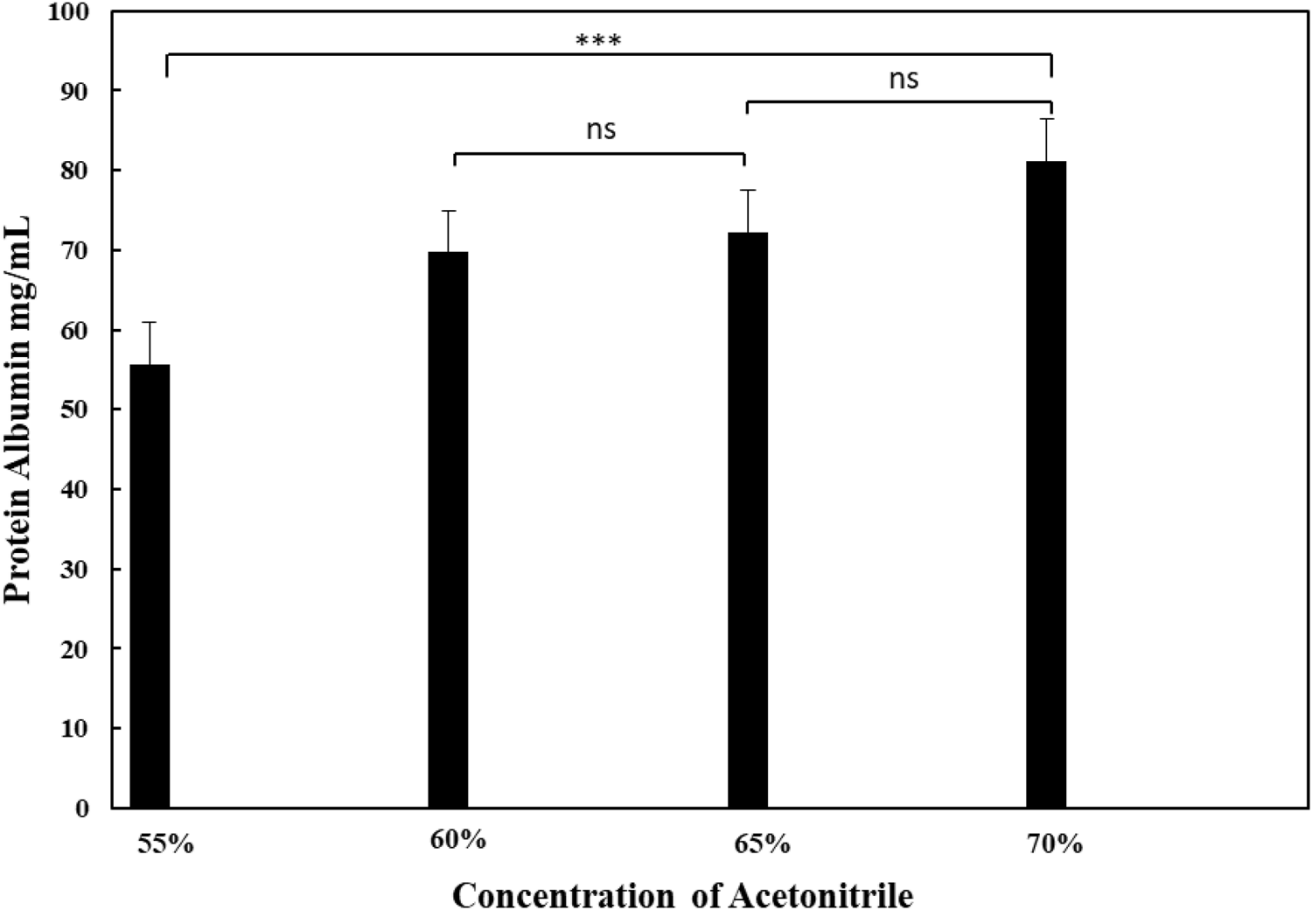
The concentration of acetonitrile solution used in the elution of albumin from the membrane (n = 3, ns = not significant, *** = p < 0.001).

Finding an appropriate concentration for eluting proteins from the membrane was performed using the membrane with the highest protein binding capacity, the PBS-imprinted ABH, and graphene oxide-composited membrane. The solvent used for protein elution is acetonitrile, which has a polarity close to that of proteins^40^. The appropriate concentration was determined by evaluating the different acetonitrile concentrations, which were selected based on the concentration that resulted in the highest protein elution. Figure 8 shows the amount of protein measured from acetonitrile with concentrations ranging from 55 to 70%. The appropriate acetonitrile concentration is 60 to 70%, with a protein yield between 70 and 80 mg/mL. However, the immersion period of the membrane in the acetonitrile solution must also be considered while determining the suitable conditions for protein elution. Therefore, the acetonitrile concentrations of 60% and 70% were selected to find the appropriate immersion time for the membrane.

**Figure 9:**
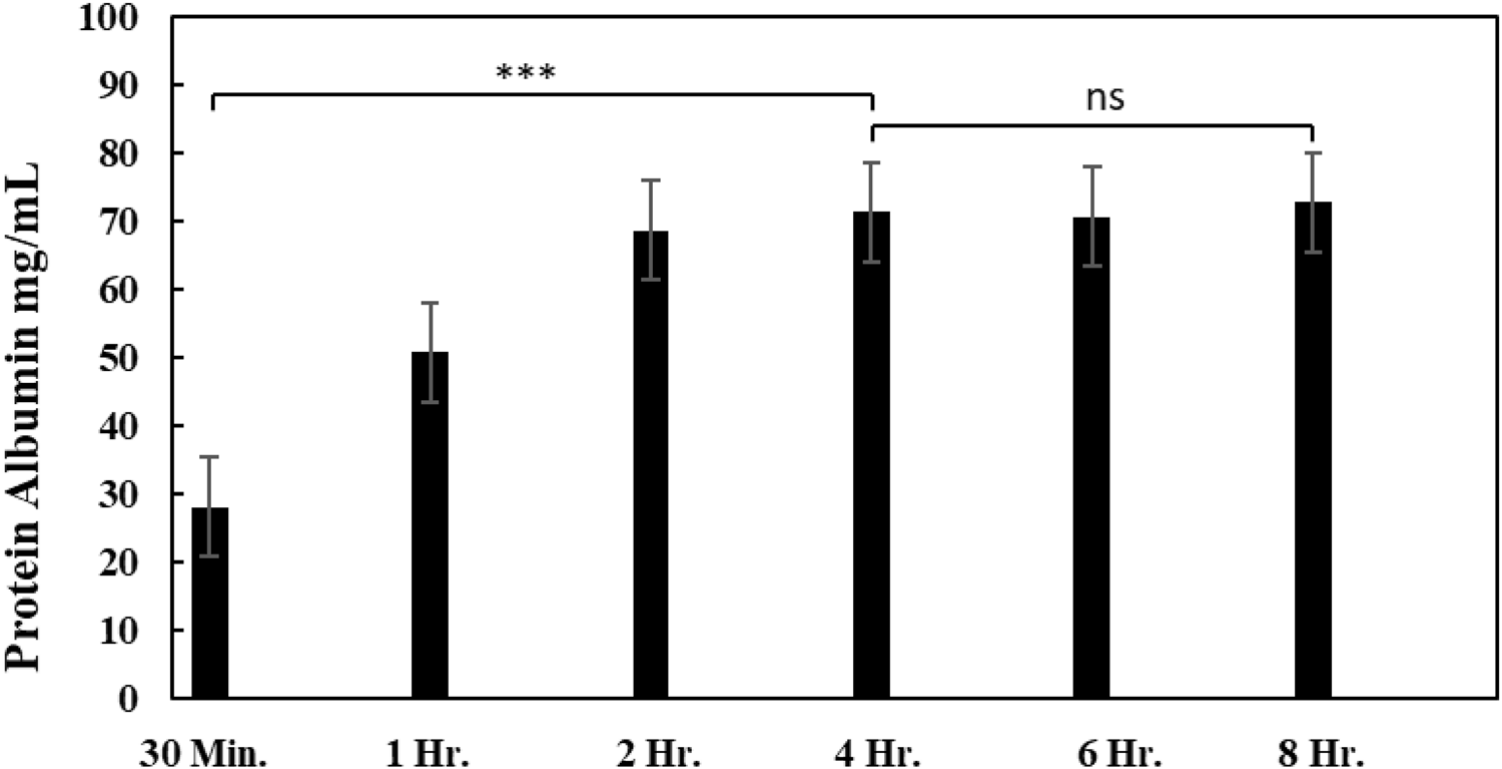
The protein content was measured for each membrane immersion period at a concentration of 60% acetonitrile (n = 3, ns = not significant, *** = p < 0.001).

**Figure 10:**
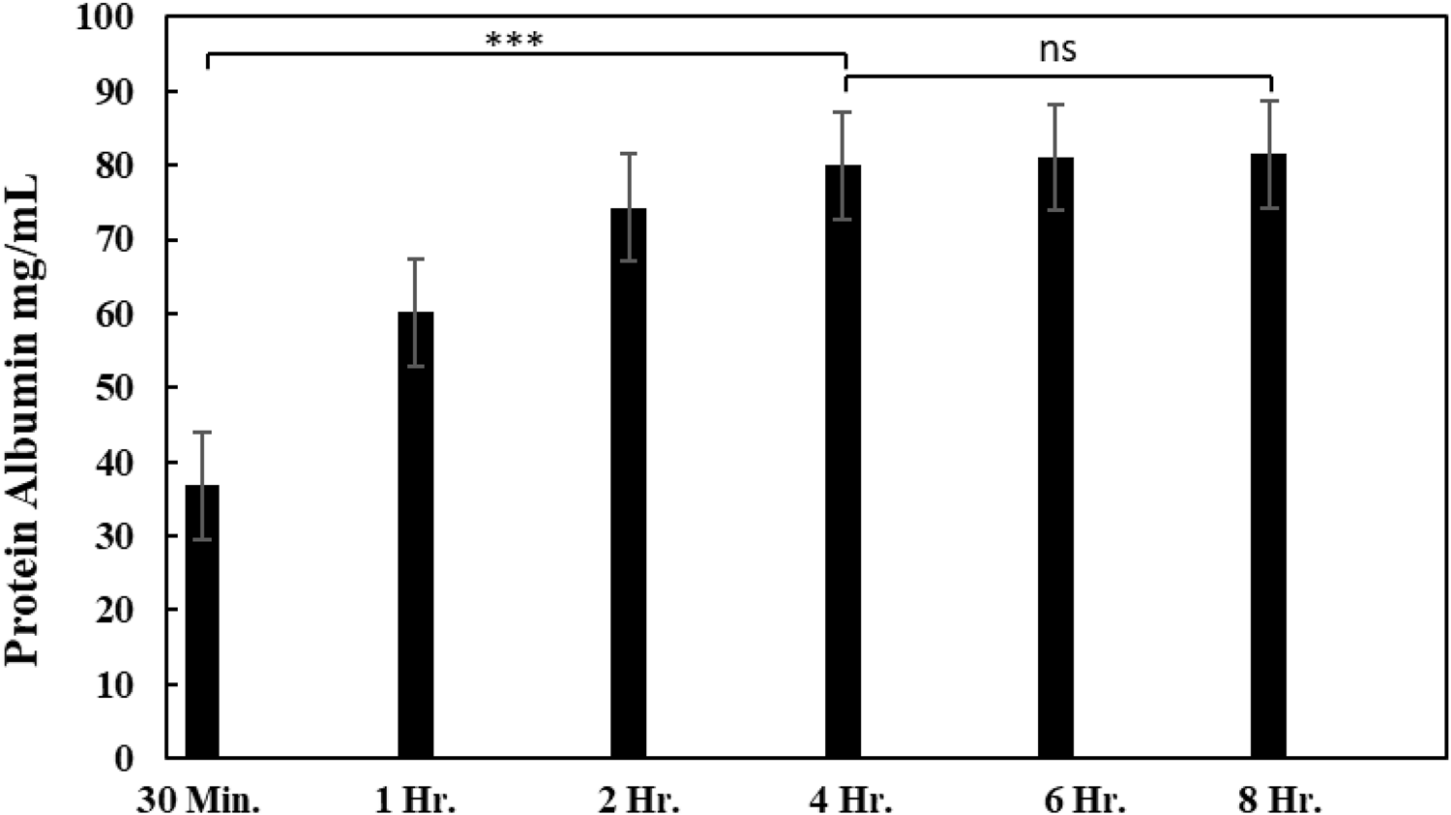
The protein content was measured for each membrane immersion period at a concentration of 70% acetonitrile (n = 3, ns = not significant, *** = p < 0.001).

The suitable immersion time was determined using the imprinting membrane of ABH and composites with graphene oxide. The immersion time was determined using the selected concentrations of acetonitrile, which were 60% and 70%. The study results on the suitable storage time are shown in Figs. 9 and 10. The study of the immersion time interval in which the concentration of 60% acetonitrile (Fig. 9) showed a maximum protein concentration of 73 mg/mL at an immersion time of 8 h. However, the measured amount of protein became constant at 4 h and beyond, with a protein concentration of 71 mg/mL, which did not differ from the protein concentration at 8 h of immersion. The study of the time range in which the membrane was immersed in 70% acetonitrile (Fig. 9) showed that the maximum protein content obtained was 82 mg/mL after 8 h of immersion. The protein content remained constant starting from the 4th hour of immersion at 80 mg/mL. The study found that the appropriate time range for both acetonitrile concentrations is 4 h, during which the measured protein quantity remains significantly stable.

In addition to developing the ability of the membrane to capture high-protein, studying methods for removing proteins from the membrane is also essential. The study shows the importance of finding an appropriate condition to prevent protein loss from the membrane during protein elution. Acetonitrile is a substance selected for protein precipitation, with the suitable concentration and time being 60% and 4 h, respectively. Although acetonitrile with a 70% concentration gives a higher protein yield than 60%, using acetonitrile with a concentration that is too high can affect the integrity of the membrane and result in protein contamination. However, the amount of protein lost from the membrane did not differ significantly from the amount captured. This study, therefore, selected a concentration of 60% as the appropriate concentration.

## Conclusions

The imprinting technique improved the protein absorption efficiency of the membrane significantly when used in conjunction with graphene oxide composites. The study showed that the synergy between imprinting and compositing with graphene oxide increased the density of the nanofibers. The increased density of electrospun fibers from using graphene oxide’s excellent electrical conductivity led to an increased surface area of the membrane. The high density of protein imprint on the nanofibers also enhanced the protein-capturing ability of the membrane. The ABH binding capability of the PBS-GO membrane could be increased from 50 to 83% by using a human albumin imprinted membrane. Additionally, the study identified the appropriate protein elution conditions to support the application of the developed membrane. The appropriate protein elution solution was 60% acetonitrile, with a 4-h immersion duration. The concentration and immersion time were adequate for protein elution while safe and non-destructive to the membrane. This research has a potential to cut down on the amount of time and the number of stages that are required for protein separation prior to further analysis.

## Data Availability

The data presented in this study are available on request from the corresponding author.
